# Prophylaxis of Childhood Migraine: Topiramate Versus Propranolol

**Published:** 2013

**Authors:** Seyed Hassan TONEKABONI, Ahad GHAZAVI, Afshin FAYYAZI, Ali KHAJEH, Mohammad Mahdi TAGHDIRI, Fatemeh ABDOLLAH GORJI, Eznollah AZARGHASHB

**Affiliations:** 1 Professor of Pediatric Neurology, Pediatric Neurology Research Center, Shahid Beheshti University of Medical Sciences, Tehran, Iran; 2 Assistant Professor of Pediatric Neurology, Pediatric Neurology Research Center, Urumia University of Medical Sciences, Urumia, Iran; 3 Assistant Professor of Pediatric Neurology, Pediatric Neurology Research Center, Hamadan University of Medical Sciences, Hamadan, Iran; 4 Assistant Professor of Pediatric Neurology, Children and Adolescents Health Research Center, Zahedan University of Medical Sciences, Zahedan, Iran; 5 Associate Professor of Pediatric Neurology, Pediatric Neurology Research Center, Shahid Beheshti University of Medical Sciences, Tehran, Iran; 6 MSc, Research Consultant, Clinical Research Development Center, Mofid Children’s Hospital, Shahid Beheshti University of Medical Sciences, Tehran, Iran; 7 Assistant Professor, Shahid Beheshti University of Medical Sciences, Tehran, Iran

**Keywords:** Migraine, Children, Propranolol, Topiramate

## Abstract

**Objective:**

Headache is a common disabling neurological disorder and migraine comprises more than half the causes of recurrent headaches in children. Despite extended prevalence of this type of headache there is lack of evidence about best drug treatment for migraine. So we aimed to compare the therapeutic effects of these drugs on childhood migraine.

**Materials & Methods:**

In the current study, a randomized clinical trial consisting of 78 patients according to 2004 International Headache Association criteria were randomly assigned to two groups that matched by age and sex. One of these two groups was treated with Topiramate, while the other was given Propranolol. After one and four months, the efficiency of these treatments was measured in terms of frequency, severity and duration of migraine attacks.

**Results:**

Results obtained from the data collected showed that of these 78 studied patients, 38 patients received Topiramate treatment (group A) and the rest (40 patients; group B) was treated with Propranolol. The average age of group A was 8.5± 2.9 years and that of group B was 8.3 ± 2.8 years. No significant difference was observed between these two groups in terms of reduction in frequency, severity and duration of migraine attacks.

**Conclusion:**

Results showed that both treatments had the same efficiency in healing migraine headaches and there was no significant difference between their treating results. However, further studies are needed to examine medical effects of these two medicines.

## Introduction

Migraine in children affects the quality of life due to its disabling qualification. It leads to absence from school; therefore, it has negative impact on the child’s school achievement and social and family life. The prevalence of childhood migraine ranges from 2.7 to 10% (1). Approximately 3-5% of school-aged children are afflicted by this entity. This ratio reaches 20% through adolescence. At lower age the predominance is towards boys eventually shifting in the direction of female gender through adolescence which remains through adulthood (2, 3).

One of the first steps of management of this disabling condition is lifestyle modifications including avoiding certain foods and habits that may have a trigger effect, medication and prevention. Some patients do not respond to these changes and need further efforts for appropriate headache control. Higher than three to four monthly headaches may be the indication to start prophylactic treatment in a patient (4). 

In 1966, Rabkin et al. accidentally recognized the impact of Propranolol for prophylaxis of migraine on patients who were under this medication for angina pectoris treatment. Anti-convulsant drugs have also been experimented for migraine prophylaxis since 1970 with carbamazepine as the first drug of this group (5).

Preventive measures which may be pharmacological or nonpharmacological are approved by some studies in case the number of headache episodes are more than three to four in a month or if the attacks are expressively as measured in the Pediatric Migraine Disability Assessment Scale (PedMIDAS) (6,7). PedMIDAS is a validated six-item questionnaire based on the adult MIDAS tool (8). It has been changed according to childhood lifestyle. The questions are concerned with the influence of headache on school performance such as whole day and partial day absences from school, 50% or less school functioning, ability to perform homework and chores and on social abilities such as sports (9,10).

For the prevention of migraine in children no drugs have been approved by the Food and Drug Administration (FDA) at the present time (11). Topiramate, an antiepileptic drug approved by FDA, has been used for the preventive treatment of migraine in adults and also the treatment of partial-onset seizures and primary generalized tonic-clonic seizures as additional therapy in children. The objective of this study was to study the prophylactic efficiency of Topiramate in childhood migraine and to compare it with Propranolol.

## Materials & Methods

This study was a randomized controlled trial that compared the efficacy of Topiramate with Propranolol in the prophylaxis of pediatric common migraine. Children aged 3-15 years with common migraine, as defined by the 2004 international headache society criteria (12), were enrolled into our study. The inclusion criteria was one of the following: 

1. More than three headaches per month 

2. Severe disabling or intolerable headache 

For all the patients who met the inclusion criteria, we recorded the duration, severity and frequency, for a period of one month before receiving the drug prophylaxis. They were evaluated in our follow up clinic after one month (first visit) and four month (second visit) for the same above mentioned headache parameters. Then these results were compared to pre-treatment state. Patients with a past trial of migraine prophylactic agent, persistent increasing headache, change of behavior, school performance, increased pain with the valsalva maneuver, abnormal physical examination (such as papilledema), persistent focal neurological signs, neuroimaging studies indicating a focal neurological lesion, contraindication for Topiramate or Propranolol (such as asthma), were excluded from the study. For the patients who entered the study, complete physical and neurological examination, baseline laboratory screening test and neuroimaging studies if necessary were performed. Consequently, the patients randomly were divided into two groups that matched by age and sex. Group A (n=44) received 50-100 mg daily Topiramate and group B (n=42) received 20-80 mg daily Propranolol, with at least 4 months follow up.

The severity of headache was ranked as follows (3). 

1. It does not affect daily activities

2. It roughly disturbs daily activities 

3. It hampers daily activities. 

Then, the data newly obtained from the groups was compared with each other and with their prior-totreatment data.

Descriptive statistics (including mean, median, range, and standard deviation, frequency and frequency percentage) were determined using statistical software SPSS 18th version. For comparison of quantitative means, independent sample t-test was used after confirmation of normal distribution of data by onesample Kolmogorov-Smirnov, repeated measurement, ANOVA, Friedman tests. The chi-square test was used for qualitative comparisons. In all tests, the significance level was considered as two tails and p <0. 05. 

As a research study this proposal acquired the Ethical Committee certificate of Pediatric Neurology Research Center.

## Results

Fourth-four patients of group A out of totally 86 patients received Topiramate and the remainder 42 patients of group B were treated with Propranolol. Six patients of group A were discarded; three patients due to drug allergy, one because of a disorder generated in educational performance and the other two patients as the result of lack of compliance. Of group B also two patients stopped being under examination; one without informing the researcher and the other due to suffering from asthma. In the Topiramate group, the average age of the patients was 8.5±2.9 years and that of the other group was 8.3±2.8 years. No significant difference was observed between these groups in terms of average age of patients (P=0.718).

Regarding the aspect of age groups, we did not find any significant difference between the two groups (P=0.890) (Table1). Nineteen patients (50%) of the Topiramate group and 21 participants (52.5%) of the Propranolol group were boys. Therefore, no significant difference was statistically found between the two groups regarding gender (P=1.0).

The data shown in Table 2 indicate comparison of average headache frequencies obtained in various visits of under examination patients suffering from migraine headache who were treated with Propranolol and Topiramate. As observed, the average frequency of headache attacks obtained from both groups in the visits during treatment shows a decrease in a way that a significant difference was generated between average frequencies of headache attacks obtained in various visits in both groups (P=0.001). These results highlight the fact that both medicines reduce the average frequency of headache attacks of patients under examination. 

Both groups showed a significant difference between the average frequency of headache attacks in pre-treatment visits (P=0.001) and those of first visits (P=0.001) and second visits (P=0.001) carried out during treatment. However, no significant difference was seen between the average frequencies of headache attacks in the first and second visits (P>0.05) between the groups during treatment.

In addition, the results revealed that from the statistical aspect, there was no significant difference between the average frequencies of attacks obtained for both groups in three visits (one held before medicine prescription and two sessions at the end of the one and four months after medicine prescription) (P>0.05). 

The data shown in Table 3 presents comparison of headache severity obtained in different visits of patients suffering from migraine headache receiving treatment with Propranolol and Topiramate. As observed, the severity of headache attacks of both groups was reduced in visits held after medicine prescription. So, a significant difference was found between the severity of headache attacks in both groups in various visits (P=0.001); demonstrating that both medicines were successful in reducing the patients headache severity of people under examination. 

However, the results showed no significant difference statistically between the two groups regarding the severity of the attacks in all visits (a visit prior to medicine prescription and two visits at the end of one and four months from medicine prescription) (P>0.05). The data shown in Table 4 compares the average duration of each headache attack which was reported in the different visits. Again, a decrease was observed in the average headache duration in both groups after the treatments were started. Additionally, a significant difference was statistically revealed between the average headache duration of various visits in each group (P=0.001); in other words, both treatments succeeded in decreasing the average duration of migraine headache in both groups. 

## Discussion

The result obtained from the present study shows that both Topiramate and Propranolol are effective in the prevention of children migraine and in reducing the number, severity and duration of migraine attacks as well. However, an effective Topiramate dosage needed to prevent migraine is less than that required to prevent epilepsy. A 50-100 mg dosage of Topiramate is of great effect in migraine prevention. However, 14% of the Topiramate-treated group stopped receiving medicine due to its side effects, while within the Propranololtreated group only one participant failed to carry on treatment as the result of asthma. 

Through a decrease generated in the severity level and duration of headache attacks, the number of days the students were absent from school was reduced and an improvement of quality of life was achieved. According to a random double blind study conducted by Lipton et al., 44 children were randomly assigned to two groups, each receiving Topiramate and placebo for a period of 12 weeks. Preliminary results were evaluated as a reduction in both attack frequency and duration and secondary results were examined as performance disability due to severe headache and reduction in number of sedatives received. The number of migraine attacks decreased in both groups some weeks after treatment. 95.2% of those treated with Topiramate revealed a more than 50% reduction in headache frequency compared to that of the placebo-treated group (51.4%). The amount of disability caused because of migraine dramatically decreased. The uppermost side effects of Topiramate in this study were reported as loss of weight (23.8%), loss of appetite (23.8%), reduction of concentration (19%) and stomachache (14.3%) (13). Our study; however, did not find any significant difference between the two groups separately receiving Topiramate and Propranolol, the averages of headache numbers and duration showed a decrease in both groups. 

Another random double blind research by Winner et al. was carried out on 131 children aged 6 to 15-year-old, receiving a 1:2 ratio of Topiramate and placebo. In the first stage, a reduction in the number of headache days was reported within the first month of follow up. The second stage which was the end of the fourth week was accompanied by a decrease in the duration of headache. The monthly average reduction of headache days in the Topiramate-treated group was more than 75% when compared to that of the control group (14%) (14). The current study also found an average reduction of two days in the headache-attack period of the group receiving Topiramate in the first visit and an average reduction of four days in the second visit, while the Propranololtreated group revealed a three-day reduction in the first visit and a two-day decrease in the second visit. 

According to Silbersteins study conducted to investigate the impact of Topiramate on migraine prevention, 30% of the participants revealed more than 60% reductions in the number of attacks occurred. Besides, the headache frequency decreased by 40-60 percent in 30% of the patients (15). The present study showed more than 60 % reduction in headache frequency in 55% of patients and 40-60 percent reduction in 24% of participants in the first follow up visit (after one month) of topiramte treatment. 

According to Goods et al, Topiramate was effective in reducing the frequency and severity level of headaches and vertigo (P=0.01) (16). Other articles argued that Topiramate could be of great effect to cure migraine, although further studies are needed to be conducted (11). In agreement with the above mentioned argument, our study found that Topiramate could be applied to control headache attacks; however, it did not find any significant difference between the impact of Topiramate and Propranolol in this regard. 

In an investigation to compare Topiramate and Propranolol, Ashtari et al. suggested that low Topiramate dosage was not only effective in migraine therapy, but it also generated few side effects and Topiramate effectiveness was greater compared to that of Propranolol [9]; however, no significant difference was observed between the treatment ability of these medications here. As previously discussed, results obtained by the current article recommend application of both medicines and confirm their curing results, but do not prefer one over the other. 

**Table 1:** Distribution of Age Groups Suffering from Migraine Headache in Two Groups

**Age Groups (years)**	**Treatment Groups**		**Total**
	**Propranolol**	**Topiramate**	
0-5	6 (15%)	7 (18.4%)	13 (16.7%)
6-8	17 (42.5%)	13 (34.2%)	30 (38.5%)
9-11	10 (25%)	10 (26.3%)	20 (25.6%)
12-15	7 (17.5%)	8 (21.1%)	15 (92.2%)
Total	40 (100%)	38 (100%)	78 (100%)

**Table 2:** Comparison of the Average Number of Headaches (in a Week/Month) between the Two Groups Before and After Treatment

Visits	Treatment Groups		P-value
	Propranolol	Topiramate	
	(range) average± SD	(range) average± SD	
Pre treatment	(2-20) 8.1±6.9	(3-20) 7.0±4.3	0.721
First visit	(0-16) 3.1±3.6	(0-16) 3.1±3.9	0.939
Second visit	(0-16) 1.8±3.1	(0-16) 2.3±4.0	0.643
P-value	<0.001	<0.001	--

**Table 3:** Comparison of Headache Severity between the Two Groups Before and After Treatment

Visits	Treatment Groups						P-value
	Propranolol			Topiramate			
	1	2	3	1	2	3	
Pre treatment	16	16	8	15	11	12	0.426
First visit	28	11	1	24	10	4	0.349
Second visit	34	5	1	32	4	2	0.797
P-value	<0.001			<0.001			-

**Table 4:** Comparison of the Average Duration of Headache atacks between the Two Groups Before and After Treatment

**Visits**	**Treatment Groups**		**P-value**
	**Propranolol**	**Topiramate**	
	**(range) average± SD**	**(range) average± SD**	
Pre treatment	(1-18) 5.5±4.0	(2-15) 5.4±3.3	0.536
First visit	(0-15) 2.9±3.6	(0-15) 2.8±3.1	0.835
Second visit	(0-15) 2.6±3.9	(0-15) 2.2±3.0	0.827
P-value	<0.001	<0.001	--

**Suggestions**

• Topiramate and Propranolol may be applied to cure migraine headache; however, decision on which one to use depends on the patient’s situation. 

• A comprehensive research needs to be designed and conducted to study Topiramate treatment for a longer period of time.
